# Novel Low-Temperature Chemical Vapor Deposition of Hydrothermal Delignified Wood for Hydrophobic Property

**DOI:** 10.3390/polym12081757

**Published:** 2020-08-06

**Authors:** Rui Yang, Yunyi Liang, Shu Hong, Shida Zuo, Yingji Wu, Jiangtao Shi, Liping Cai, Jianzhang Li, Haiyan Mao, Shengbo Ge, Changlei Xia

**Affiliations:** 1 Co-Innovation Center of Efficient Processing and Utilization of Forest Resources, College of Materials Science and Engineering, Nanjing Forestry University, Nanjing 210037, Jiangsu, China; yangrui@njfu.edu.cn (R.Y.); liangyunyi@njfu.edu.cn (Y.L.); hongshu.320@163.com (S.H.); zuoshida@163.com (S.Z.); wuyingji@njfu.edu.cn (Y.W.); shijt@njfu.edu.cn (J.S.); liping.cai@unt.edu (L.C.); maohaiyan@njfu.edu.cn (H.M.); 15952033445@163.com (S.G.); 2 China Jiangsu Key Open Laboratory of Wood Processing and Wood-Based Panel Technology, Nanjing 210037, Jiangsu, China; 3 Department of Chemical and Biomolecular Engineering, University of California, Berkeley, CA 94720, USA

**Keywords:** low-temperature chemical vapor deposition (CVD), hydrophobic, wood, dichlorodimethylsilane, polydimethylsiloxane (PDMS)

## Abstract

As a hydrophilic material, wood is difficult to utilize for external applications due to the variable weather conditions. In this study, an efficient, facile, and low-cost method was developed to enhance the hydrophobicity of wood. By applying the low-temperature chemical vapor deposition (CVD) technology, the polydimethylsiloxane-coated wood (PDMS@wood) with hydrophobic surface was fabricated employing dichlorodimethylsilane as the CVD chemical resource. The result of water contact angle (i.e., 157.3°) revealed the hydrophobic behavior of the PDMS@wood. The microstructures of the wood samples were observed by scanning electron microscopy and energy dispersive X-ray spectroscopy (EDS) analysis verified PDMS successfully coated on wood surfaces. The chemical functional groups of the PDMS@wood were investigated by Fourier transform infrared (FT-IR) and Raman spectra. The thermogravimetric results indicated the enhanced thermal stability of the wood after PDMS coating. In addition, the stability test of PDMS@wood indicated that the hydrophobicity properties of the PDMS@wood samples were preserved after long-time storage (e.g., 30 days). The scratch test was carried out to examine the abrasion resistance of the hydrophobic coatings on PDMS@wood surface. It was suggested that low-temperature CVD process could be a successful approach for fabricating hydrophobic wood.

## 1. Introduction

Wood is a low-cost and abundant forest biomass material, which plays a dominant role in the proper performance in every field, e.g., furniture, artworks, buildings, etc. [1]. It is generally known that wood is multifunctional, environmentally friendly, and a renewable resource with numerous advantages, for examples, high strength-to-weight ratio, high impact resistance, high processability, and the regulation function of air temperature and humidity [2,3,4]. The environment impact of natural materials (for examples, flax fibers and wood) is lower than that of glass or carbon composites [5,6,7]. However, due to the hygro-expansion caused by moisture absorption, wood is highly dimensionally unstable, restricting its wide application [8]. Therefore, the design of a hydrophobic surface is very necessary for the further applications [9,10]. For the enhancement of dimensional stability, the wood surface can be modified by the hydrophobic treatment to reduce the wood sensitivity with the change of environmental humidity and the risk of wood moisture absorption [11].

Numerous studies have been conducted on the modification of wood for improving its hydrophobicity. It can be summarized into three approaches to improve the hydrophobic, for instance, improving wood surface roughness, using low surface energy material, and stuffing the woody cells and vessels [12,13]. According to the properties of the hydrophobic treatment method, it can be divided into physical and chemical methods. The physical method included the plasma etching [14], spraying [15], electrostatic layer-by-layer assembly [16], etc. The chemical modifications mostly included the sol-gel process [17], hydro-thermal method [18], and chemical vapor deposition [19,20].

Poplar is a quick-growing species of trees with a short growth cycle and low price [21]. With straight wood texture, uniform structural, good dimensional stability, and softness, poplar is widely selected to be the experiment material. Its basic density of poplar is between 17.39 kg/m^3^ and 18.60 kg/m^3^ [22]. From the previous study, poplar had many internal spaces, in which the overall porosity of poplar is accounted for approximately 49% [23]. Thus, poplar wood was used as the experimental sample due to its natural advantages.

However, many modification methods cannot be widely applied because of the complex preparation process, high cost, and long production cycle. The low-temperature chemical vapor deposition (CVD) was utilized to the hydrophobic modification for the cellulose-based materials and wood composite materials. For example, Gamelas et al. [24] implemented fast CVD to fabricate hydrophobic pulp by using trichloromethylsilane vapor silanization. Although this method could offer the hydrophobic function to the modified surfaces by the salinization treatment, the size of cellulose fibers was in the nanometer range.

The functionalization of natural solid wood can be achieved using PDMS via low-temperature CVD reaction, which generated a hydrophobic material with apparently unique surface characteristics. However, in the literature review, no report regarding the utilization of this technology for wood-based materials was found. Therefore, the design and fabrication of hydrophobic surface on wood with stable interaction by low-temperature CVD is worthy of being investigated.

This study was aimed at developing hydrophobic wood with high abrasion resistance by poplar. To promote the gas penetrating into wood cells, the poplar wood samples were pretreated to change the structure of wood cells. Dichlorodimethylsilane gas and water vapor were injected to wood cells to produce polydimethylsiloxane (PDMS), which was firmly fixed onto the surfaces of inside of the wood cell walls and vessel walls. After this process, the stabilized hydrophobic characteristics of PDMS@wood samples were obtained.

## 2. Materials and Methods

### 2.1. Materials

Poplar (*Populus* spp.) obtained from the state-owned Jiaozuo forest farm (Henan, China) was chosen as raw materials, which was cut into the size of 20 mm × 20 mm × 3 mm (Longitudinal × Tangential × Radial) for experiments. Dichlorodimethylsilane (C_2_H_6_Cl_2_Si), sodium bisulfite (NaHSO_3_), sulfuric acid (H_2_SO_4_), and calcium sulfate (CaSO_4_) from Aladdin (Shanghai, China) were of analytical grade and used as received. Deionized (DI) water was used in the experiment. Four groups of samples including cross section of untreated wood, tangential section of untreated wood, cross section of PDMS@wood, and tangential section of PDMS@wood were prepared and tested.

### 2.2. Dichlorodimethylsilane Vapor Deposition

**Figure 1:**
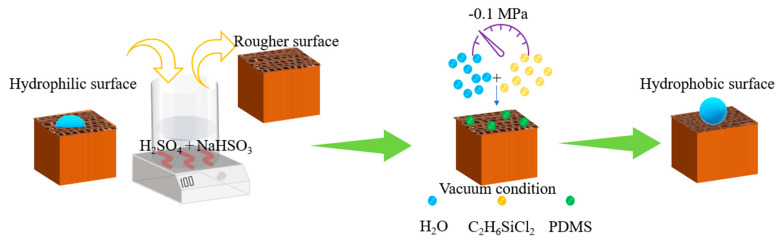
Schematic illustration of description of the entire process.

The schematic illustration of description of the entire process is presented in Figure 1. Pretreatment was carried out to weaken the bindings among cellulose, hemicellulose, and lignin, so that the dichlorodimethylsilane vapor could easily penetrate wood cells in this research. In the pretreatment process, the wood samples were dipped in the dilute H_2_SO_4_ solution (0.4 vol.%) and followed by adding NaHSO_3_ (10 wt.% of wood) into the solution. After properly stirring, the mixture was heated to 100 °C and dwelled for 20 min using a water bath with thermocouple. Then, after the samples were removed and washed by DI water, they were oven dried for 2 h at approximately 100 °C. It was observed that the sample surfaces were become rougher owing to the damaged cell walls during the pretreatment. Finally, the samples were sanded by sandpaper for improving the surface flatness.

The reaction device consisted of two concatenated flasks of different specifications. The pretreated woods were placed into the reaction device, and the reactor was vacuumed to −0.1 MPa, and kept for 15 min to allow the gas to penetrate into wood cells easily due to the pressure difference between the environmental condition and the pre-vacuumed internal wood cells. The flask connecting between the DI water and the reactor played a key role as an anti-suction flask. Both valves should be closed before DI water was heated to 60 °C. As the water was heated, the inlet gas flow ensured the uniform particles to deposit over the entire surface of the substrate. When the heating temperature reached the target temperature, the left valve was opened, and the DI water vapor was fed into the reaction device for 10 min. After that, the dichlorodimethylsilane gas was diffused into the reaction flask with the opening of the right valve. It should be noticed that the dichlorodimethylsilane needed to be heated to 30, 50, and 70 °C without the opening of the offside valve, and the temperature of each reaction should be maintained for 0.5, 1, and 1.5 h as control groups. Dichlorodimethylsilane with moisture went to dimethyldihydroxysilane because of the hydrolysis reaction. Dimethyldihydroxysilane was further polymerized to produce PDMS@wood surface, which made the poplar samples to have hydrophobicity. After being dried at 100 °C for 2 h, the PDMS@wood sample was prepared.

### 2.3. FT-IR and Raman Analysis

Infrared analyses were performed using the Fourier transform infrared spectrometer (Nicolet380 FT-IR, Waltham, MA, USA) with Attenuated Total Reflectance (ATR) module. The scan rage was 4000 to 700 cm^−1^, the resolution was 4 cm^−1^, and 16 scans were taken for each data point. In this test, the cross section and tangential section of PDMS@wood samples processed at reaction temperature of 70 °C and reaction time of 1 h were compared to the untreated wood samples. The infrared spectra of the samples were tested and analyzed by the OMNIC software. Raman spectra were recorded on a Thermo DXR 532 (Waltham, MA, USA) FT Raman spectrometer with laser excitation at 780 nm. All spectra were recorded in the range of 3300 to 50 cm^−1^, with a nominal resolution of 4 cm^−1^.

### 2.4. TG Analysis

The thermogravimetric (TG) analysis was performed (TGA55, TA Instruments, Milford, MA, USA). The initial wright of TG sample for untreated wood and coated wood was maintained at approximately 7 to 9 mg. The experimental samples were ground into approximately 100 mesh sizes. The analysis was run in the nitrogen flow from 30 to 800 °C, with ramping at 5 °C/min. Triplicate was taken for each measurement, and the average weight loss and derivative curves were recorded.

### 2.5. Hydrophobicity Measurement

The hydrophobic property of the PDMS-coated wood was evaluated by water static contact angle (CA) and dynamic sliding angle (SA). The water CA of the modified wood surface was measured by the Theta optical contact angle measuring instrument (JC2000C POWEREACH, Shanghai, China). The experimental reaction temperatures were 30 °C, 50 °C, and 70 °C, and each reaction temperature was maintained for 0.5 h, 1 h, and 1.5 h. In each experimental condition, 4 samples were selected, among which two test surfaces were cross sections and the others were tangential sections. All samples were completely dried before the tests, and each sample was tested at five different locations including the center and four corners of the samples. The static CA was recorded 100 s after a water droplet (5 µL) was placed on the sample surface. The SA was determined by the minimum tilt angle at which a water droplet (10 µL) rolls off the surface. The data of CA were collected at the time durations of 0 s (Initial contact angle), 20 s, 40 s, 60 s (Equilibrium contact angle), 80 s, and 100 s to describe the hydrophobicity of the samples. All the data acquisitions were based on the droplet staying time on the sample surface.

### 2.6. Surface Observation

The surface morphology was characterized by the scanning electron microscopy (Quanta 200 SEM, Hillsborough, OR, USA). The cross and tangential section of PDMS@wood samples processed at the reaction temperature of 70 °C for 1 h were compared to the untreated wood. The samples were severally cut into the sizes of 2 × 2 × 3 mm (Longitudinal × Tangential × Radial) and 2 × 2 × 3 mm (Radial × Tangential × Longitudinal) by reprocessing. The samples were glued to the object stage, then coated with gold for 6 s after the vacuum pump (10^−1^ Pa) with an Ion Sputter Coater (SCD005, Leica, Wetzlar, German).

### 2.7. Surface Abrasion Resistance Test

To quantify the mechanical stability of the treated sample, an abrasion test was conducted in this study. The sandpapers (1500 mesh) were purchased from Alibaba (Hangzhou, China). The surface of hydrophobic wood was in close contact with sandpaper and moved in one direction with a constant external force and speed under the condition of applying 100 g weight pressure. This process was one cycle, and a total of 10 cycles of wear resistance tests were carried out to simulate the most serious condition in real-life. The water CAs and SAs of PDMS@wood surface after the abrasion test were also measured by the Theta optical contact angle measuring instrument (JC2000C POWEREACH, Shanghai, China) and rolling angle test.

## 3. Results and Discussion

### 3.1. Surface Dichlorodimethylsilane Vapor Deposition

**Figure 2:**
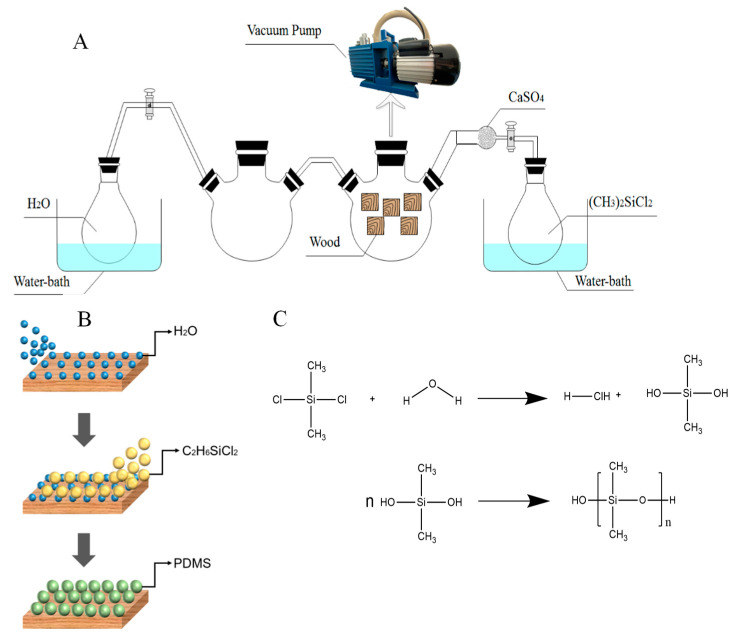
Low-temperature chemical vapor deposition (CVD) treatment of wood. (**A**) The device set-up of the treatment and (**B**) illustration of the formation of PDMS on the wood surface, including (I) moisture deposition onto the wood surface, (II) introducing C_2_H_6_SiCl_2_ vapors onto the wetted wood surface, and the (III) the formation of PDMS though the (**C**) chemical reactions.

The flowchart for preparing the hydrophobic wood is shown in Figure 1. The wood cell wall, including cellulose and hemicellulose, was destroyed by the pretreatment. Then, the wood samples were vacuumed to facilitate hydrophobic gas to enter the wood for reaction. Therefore, the prepared hydrophobic wood not only had a hydrophobic layer on the surface, but also had a better result than the samples without the pretreatment because the gas more easily penetrated into the pore structures of the wood compared with the solution. Figure 2A schematically illustrates the procedure for preparing the hydrophobic coating. Figure 2B,C illustrates the construction principle of hydrophobic wood. It was suggested that the roughness of PDMS particles endowed the hydrophobicity property to the PDMS@wood [25]. This technology does not affect the wood properties, unlike some methods which can only modify a single property.

**Figure 3:**
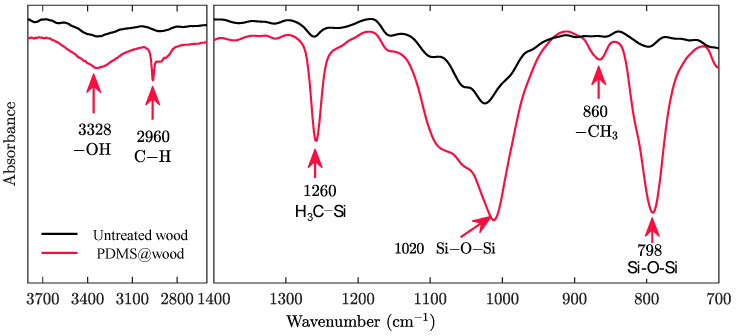
FT-IR spectra of untreated wood and PDMS@wood.

FT-IR analysis was applied to further understand the chemical structural changes and analyze some characteristic bands of the untreated wood and PDMS@wood. The FT-IR spectra are shown in Figure 3. The broad and intense band at 3050–3700 cm^−1^ with its center at approximately 3328 cm^−1^ corresponds to the O–H stretching vibrations of the surface hydroxyl groups and adsorbed water [26]. The polydimethylsiloxane chain possesses 2 Si–OH groups judging from the peak of C–H stretching of the CH_3_ groups in PDMS was observed at 2960 cm^−1^ and the characteristic of methyl silanes at 1260 cm^−1^ due to the C–Si stretching of the CH_3_–Si groups, which were important clue to the PDMS@wood successfully [27]. As depicted in Figure 3, there was an enhanced band at 1020 cm^−^^1^, which was attributed to the asymmetric in Si–O–Si stretching illuminated PDMS molecules formed a helical structure [28]. In addition, the characteristic peak of 798 cm^−1^ was obviously strengthen due to the Si–O–Si bending [29]. These results of the FT-IR spectra confirmed that PDMS coated on wood successfully.

**Figure 4:**
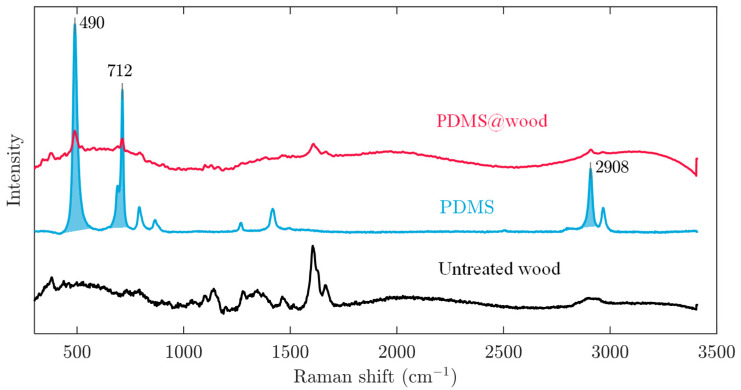
Raman spectra of the untreated wood, PDMS, and PDMS@wood.

Raman spectra were also obtained to further illustrate how the PDMS interconnected to the wood surface. The Raman spectra of untreated wood, pure PDMS, and PDMS@wood samples are shown in Figure 4. A typical Raman spectrum of the pure PDMS elastomer material showed the following peaks characterizing the various chemical bonds in the polymer; 490 cm^−1^ (Si-O-Si symmetric stretching), 712 cm^−1^ (Si-C symmetric stretching), 787 cm^−1^ (CH_3_ asymmetric rocking and Si-C asymmetric stretching), 859 cm^−1^ (CH_3_ symmetric rocking), 1262 cm^−1^ (CH_3_ symmetric bending), 1411 cm^−1^ (CH_3_ asymmetric bending), 2908 cm^−1^ (CH_3_ symmetric stretching), and 2908 cm^−1^ (CH_3_ asymmetric stretching) [30,31,32]. The pronounced peaks originating from Si-O-Si symmetric stretching at 490 cm^−1^, Si-C symmetric stretching at 712 cm^−1^, and CH_3_ asymmetric stretching at 2970 cm^−1^ observed on the PDMS@wood surface were attributed to the successful adhesion of the PDMS to the wood surface. The formation of inorganic products (silicon and carbon) clearly verified that the treatment process caused chemical transformations, i.e., silicone decomposition on the wood surface. This chemical activation of the wood surface was expected to significantly influence the water repellency of the wood surface because of the formation of low-surface energy species on the wood surface.

**Figure 5:**
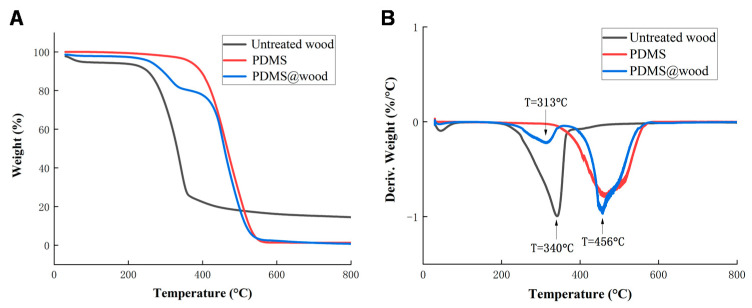
Thermogravimetric curves of untreated wood, PDMS, and PDMS@wood: (**A**) TG and (**B**) DTG.

The thermal decomposition characteristics of the untreated wood samples, pure PDMS, and PDMS@wood are presented in Figure 5. The weight loss of the untreated wood began at ~200 °C where the hemicellulose component began to decompose (Figure 5A). Moreover, the rate of weight loss increased rapidly above 200 °C as hemicellulose decomposed, until approximately 300 °C. The rate of weight loss increased again above 300 °C, which was due to the decomposition of cellulose and lignin reaching its maximum at 350 °C. At 400 °C, the lignin decomposition continued with heat treatment up to 800 °C, yielding solid carbon [33]. Then, above 800 °C, there were no significant mass changes in all samples. The residual mass losses of the untreated wood, PDMS, and PDMS@wood after TG tests were approximately 14.47%, 6.82%, and 8.94%, respectively. The initial weight loss PDMS@wood samples were 4.5–5% (Figure 5A), because of the evaporation of free water [33]. Then, the first sharp mass loss of almost 80% occurs in the temperature range of 300 to 420 °C for the untreated wood samples, and between 200 to 600 °C for the PDMS@wood, respectively. More complex decompositions happened from approximately 370 to 600 °C. It showed that higher thermal stability was achieved after PDMS coating on wood surface. The starting temperature of the thermal degradation of PDMS increased from 300 to 400 °C confirming the removal of low molecular weight oligomers [34].

As seen from the DTG plot in Figure 5B, the mass loss of PDMS@wood at the first region range (220–350 ℃) and in the second (350–600 °C) can be assumed as the amounts of the degradation of hemicellulose and PDMS, respectively [34]. Degradation of PDMS was remarkable at 600 °C with a mass loss of 38%, being ascribed to the deprivation of methyl groups in Si-O backbone [35]. The remaining materials were mainly solid carbon and ash [36]. PDMS thermal degradation of the depolymerization pathway occurred through two competing mechanisms including unzip degradation and rearrangement degradation [37]. Both degradations happened at more than 400 °C after the degradation of cellulose, hemicellulose, and lignin of natural wood. In contrast with the untreated wood samples, the characteristic peaks were concentrated at 340 and 440 ℃ from the DTG curves of the PDMS@wood samples (Figure 5B). The cellulose and lignin of natural wood were degraded at the range of 220 to 360 °C. Moreover, the cyclic siloxanes of different dimensions were generated due to PDMS degradation at the range of 360 to 500 °C [38]. When the temperature exceeded 500 °C, PDMS molecules underwent rearrangement degradation by the heterolytic cleavage. The rearrangement of Si-O-Si bond in the main chain produced low molecular weight species and rings [38]. After the PDMS coating was added onto the wood surface, the first decomposition temperature of PDMS@wood was approximately at 313 °C, ascribed to the degradation of wood chemical composition. Then, the second degradation proceeds via the depolymerization pathway occurring at 456 °C with the PDMS unzip degradation. These findings demonstrated that the PDMS was successfully coated onto the wood.

### 3.2. Microtopography

**Figure 6:**
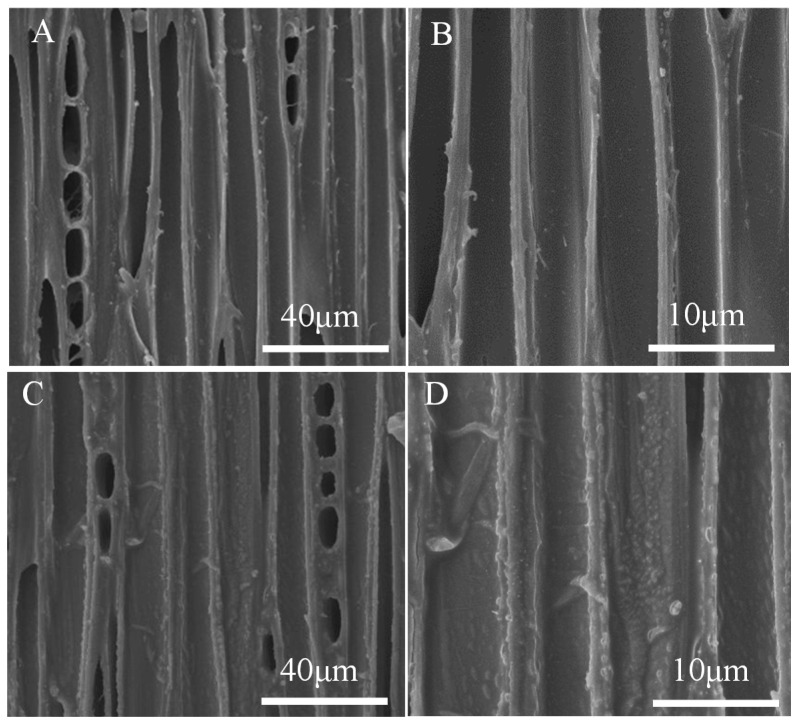
The SEM images of wood samples on tangential section: (**A**,**B**) untreated wood; (**C**,**D**) PDMS@wood.

**Figure 7:**
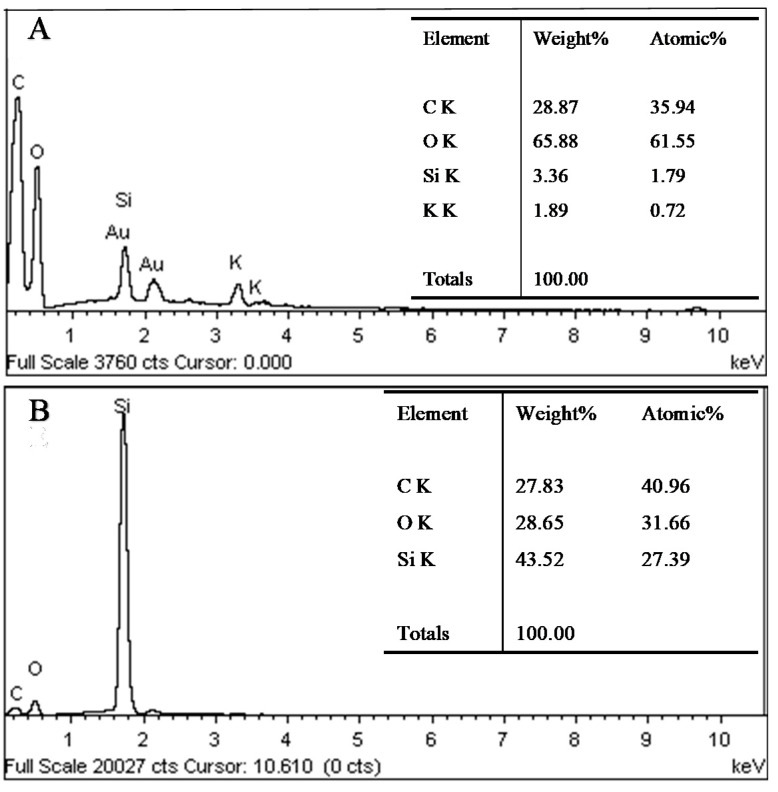
The EDS analysis of wood samples on tangential section (**A**) untreated wood and (**B**) PDMS@wood.

The influence of PDMS deposition of wood samples on the tangential surface was investigated by the SEM analysis shown in Figure 6. On the tangential section, the wood vessel wall of untreated wood was smooth and distinct (Figure 6A,B). The PDMS@wood cell wall showed a particulate morphology on the surface (Figure 6C,D), which provided high roughness to play a significant role in forming the hydrophobic surface. The granular and particulate materials were silicon from PDMS, which was verified by the EDS analysis of the PDMS@wood samples (Figure 7B). Although the PDMS@wood samples were processed by low-temperature CVD, no visible damage of the wood microstructure was found. The particulate part was random distribution on the wood cell walls. Combining with the FT-IR and EDS analysis results, the wood cell walls were successfully covered with PDMS after the CVD modification. Indeed, the adequate thickness of PDMS hydrophobic coating existed in wood surfaces, walls, and cavities. The EDS spectra of the wood samples on tangential surface for elemental analysis are shown in Figure 7. The untreated wood was mainly composed of C (28.87%) and O (65.88%) elements and trace amounts of Si elements (3.36%) (Figure 7A). The presence of a significant Si peak at ~1.8 keV in the spectra of the PDMS@wood sample with 43.52% weight ratio (Figure 7B) suggested that PDMS was successfully grown on the wood surface through the CVD process.

**Figure 8:**
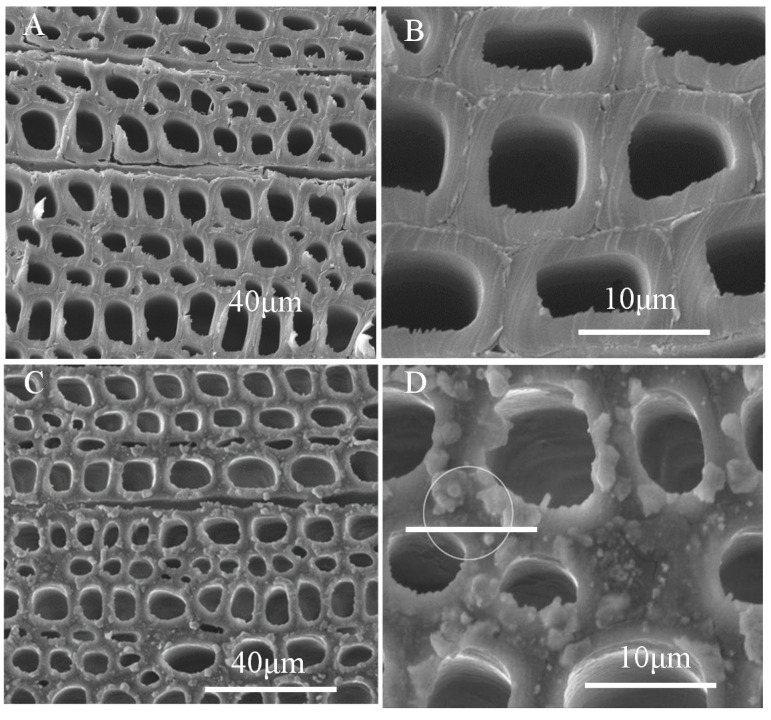
The SEM images of wood samples on cross section: (**A**,**B**) untreated wood; (**C**,**D**) PDMS@wood.

**Figure 9:**
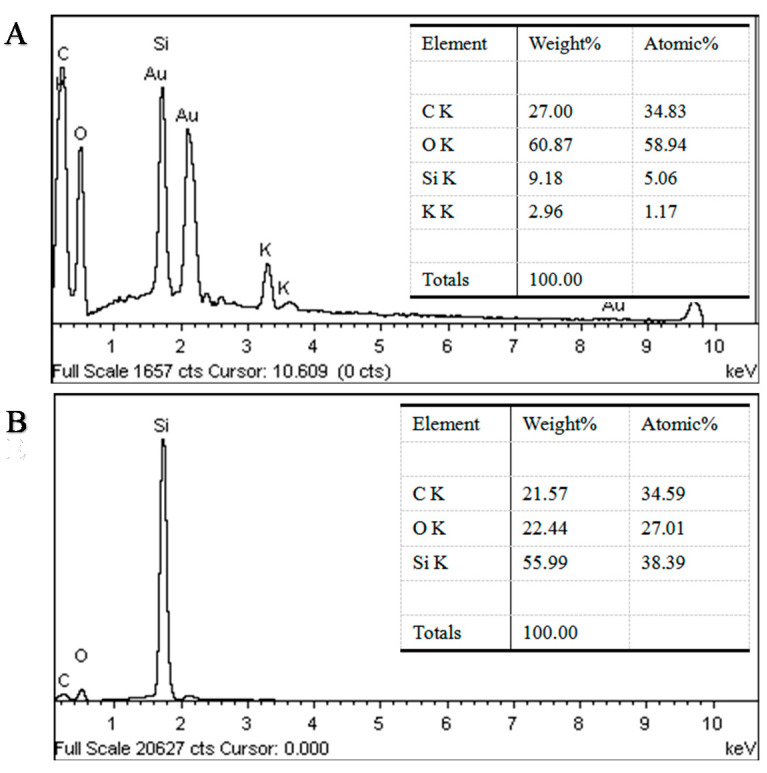
The EDS analysis of wood samples on cross section (**A**) untreated wood and (**B**) PDMS@wood.

The SEM images of wood samples on cross section showed the perfect ellipse shape of the pore, with uniform shapes and flat surfaces (Figure 8A,B). There were massive lumens with a typical diameter of approximately 20 µm. The particles, with different size and shape, were found in the surface of the PDMS@wood samples. As displayed in Figure 8A,B, the untreated wood surface was glossy smooth except uneven cutting. As depicted in Figure 8C,D, it was observed fluorescent substance of the tracheid wall, which could be the PDMS molecule. The EDS spectra of the wood samples on cross section are shown in Figure 9 to evaluate the content of elements on different surfaces. The untreated wood was mainly composed of C (27%) and O (60.87%) elements and trace amounts of Si elements (9.18%) (Figure 7A). The presence of a significant Si peak at ~1.8 keV in the spectra of the PDMS@wood sample with high weight ratio of 43.52% (Figure 7B) suggested that more PDMS coated on the wood cross surface through the CVD process. This result coincided with the following hydrophobicity analysis of PDMS@wood on different surfaces of wood that the cross section of PDMS@wood possesses better hydrophobicity.

### 3.3. Effect of Dichlorodimethylsilane Vapor Deposition on the Surface Hydrophobicity of PDMS@wood

**Figure 10:**
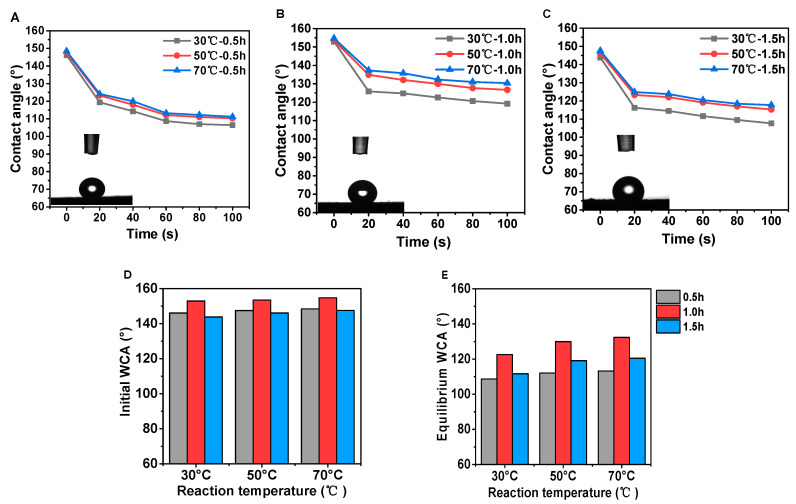
The water CAs of PDMS@wood on cross section. The different reaction temperature with durations of (**A**) 0.5 h, (**B**) 1 h, and (**C**) 1.5 h. The comparison of CAs at (**D**) initial and (**E**) equilibrium after 60 s.

Water CAs greater than 90° denote hydrophobic surfaces (non-wettable), whereas those that are smaller than 90°, represent hydrophilic ones [39]. The plot of the water CA for the samples treated by low-temperature CVD treatment is shown in Figure 10. The initial maximum water CA can reach 156.0°, which means conspicuous hydrophobic property on cross section of PDMS@wood. Besides, it was observed that, with the increase of temperature the water CA curve improved at the same time condition, 70 °C was the ideal reaction state (Figure 10). The best initial CA was achieved in 0.5 h treatment at 70 °C to be approximately 148.5°, which gradually dropped to 111.2°. After heating treatment at 70 °C for 1 h, the maximum water CA was increased to 156.0° (Figure 10B). However, the initial water CA decreased to 147.6° in 1.5 h treatment at the same temperature (Figure 10C). The initial water CA showed that the good effect on water-resistant after the modification. The CA at 60 s was considered as the equilibrium CA of distinguish superiority in this study (Figure 10D,E). When the reaction time was 1 h at 70 °C, the water CA decreased slowly comparing with others. It was therefore concluded that the processing time with 1 h at 70 °C was the optimal condition to endow the samples with excellent water-repellent properties.

**Figure 11:**
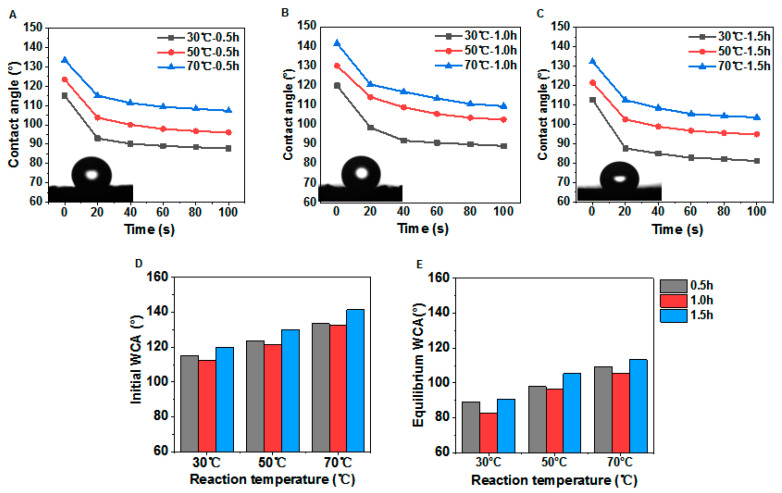
The water CAs of PDMS@wood on tangential section. The different reaction temperatures with durations of (**A**) 0.5 h, (**B**) 1 h, and (**C**) 1.5 h. The comparison of CAs at (**D**) initial and (**E**) equilibrium after 60 s.

The influence of different treatment temperatures on the water CA of the tangential section of poplar samples after the modification was shown in Figure 11. The initial maximum water CA can reach 143.0° when reaction time was 1 h, which means obviously hydrophobic property on cross section after low-temperature CVD treatment with suitable reaction time (Figure 11B). Moreover, with the increase of reaction temperature, the CA enhanced significantly (Figure 11A–C). As shown in Figure 11D,E, the reaction time had a great influence on water CA as well. The initial water CA and the equilibrium water CA differed by 20° at the same reaction temperature. It was indicated that a higher treatment temperature had conspicuous effects, which were consistent with the above CA results in Figure 11A–C. In general, the water CA for PDMS@wood samples were significantly higher compared to the untreated samples, which indicated that the low-temperature CVD method was effective to increase the wood hydrophobicity.

**Figure 12:**
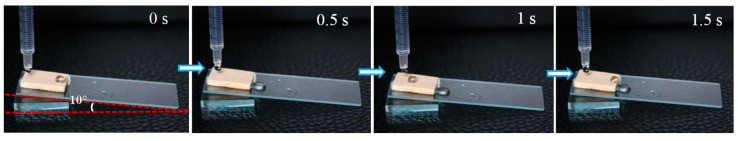
The dynamic SA test of PDMS@wood.

The dynamic sliding angle (SA) was investigated to examine the hydrophobicity of the PDMS@wood surface. The spherical water droplets rested steadily on the hydrophobic wood PDMS@wood surface, which contrasts with the instant penetration of water into the untreated wood. The PDMS@wood surface was not only hydrophobic but also showed self-cleaning properties, which was demonstrated by dynamic SA of dropping water (Figure 12). When dropped on the slightly tilted surface, the continuous water droplets readily rolled off the hydrophobic wood surface (see Video S1). The slightly tilt was ~10 degrees and indicated excellent hydrophobicity.

### 3.4. Investigation of the PDMS@wood after Long-Time Storage

**Figure 13:**
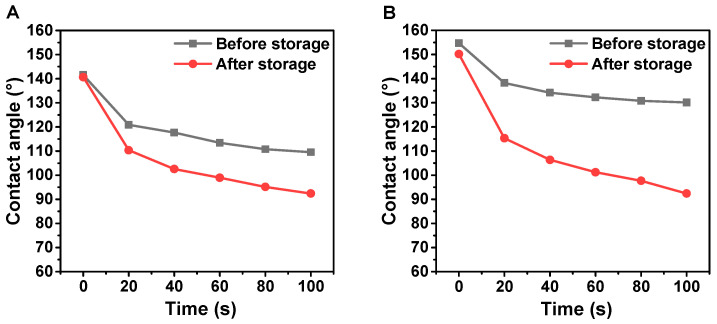
(**A**) Poplar cross section CA after 30-day storage. (**B**) Poplar tangential section CA after 30-day storage.

The tangential section samples of poplar at reaction temperature of 70 °C for treatment time of 1 h were selected for the comparison of water CA changes after storing for 30 days. Figure 13 shows the variation of the water CAs with time. The water CA of samples after 30-day storage was still 140.7°, which was just lower than the maximum static water CA of 141.5° before storage. It was indicated that the hydrophobic surface was still stable after 30-day storage. When the DI water droplet stayed for 100 s on poplar tangential section without storing, the water CA was changed (down 22.6%). Although the water CA was reduced by 34.3% after the 30-day storage, it was considered that the treatment had good effect of hydrophobicity on the samples surface.

With the same of reaction condition, the samples of poplar cross section were chosen in this experiment. Comparing with the samples without storage, the samples of the poplar cross section after long-time storage had the advantage of maintaining hydrophobicity. A structure was denoted as a “superhydrophobicity structure” when the initial water CA of the sample reached more than 150° [40]. When the DI water droplet stayed for 100 s on poplar cross section without storing, the water CA was slightly decreased to 2.9%, and the water CA was up to 130° at 100 s. It was clearly indicated that the samples still had good effect of hydrophobicity even after long-time storage. This result implied that PDMS@wood had a highly stable hydrophobic structure after long-time storage.

### 3.5. Abrasion Resistance of the PDMS@wood

**Figure 14:**
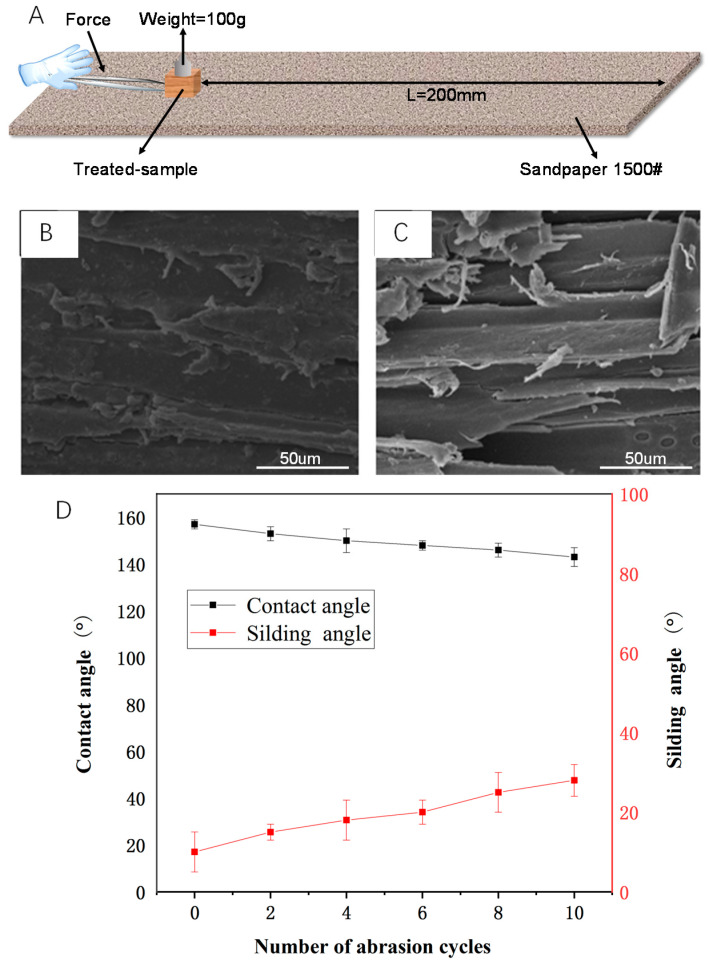
Abrasion resistance of PDMS@wood. (**A**) Schematic of the illustration of the sandpaper abrasion test. (**B**) The SEM image of the PDMS@wood surface before abrasion test. (**C**) The SEM image of the PDMS@wood surface after abrasion test. (**D**) The water CA and SA as a function of number of abrasion cycles for hydrophobic wood surface.

In general, hydrophobic coatings are often mechanically and chemically weak against damages during real-life use [41]. To quantify the mechanical stability of the hydrophobic wood surface, the sandpaper (1500 mesh) friction test was used to evaluate the abrasion resistance of wood surface hydrophobic coating in this study (Figure 14A). Before the sandpaper abrasion test, lots of particulate matter was adhered on the wood cavity from the examination of the tangential section using the scanning electron microscope (Figure 14B,C). The particulate matter belonged to the PDMS hydrophobic coating. The microstructure of the hydrophobic surface of wood was slightly destroyed, and the hydrophobic coating still could be observed after the abrasion test. It was clearly revealed that the PDMS@wood surface remained hydrophobic even after the severe abrasion.

The major issue for the practical application of hydrophobic wood is that the elaborately fabricated microstructures of the rough surfaces can be easily damaged by mechanical abrasion [42]. The scratch test was carried out to examine the abrasion resistance of the hydrophobic coatings on the PDMS@wood surface. The changes in CAs and SAs with 10 times abrasion cycles for the PDMS@wood surface are shown in Figure 14D. The CAs of the PDMS@wood remained constant at ~150° and all above 140° after being scratched repeatedly, indicating abrasion resistance property on PDMS@wood surface. At the same time, the mechanical abrasion caused a corresponding increase in the SAs, showing that the microscale structures on the PDMS@wood surface were damaged after the scratch test, whereas the nanoscale features of the silica hybrid from the PDMS coating in the cell lumens were well retained.

## 4. Conclusions

In summary, hydrophobic wood with excellent water resistance and mechanical stability was successfully fabricated by the low-temperature CVD of PDMS. The modified wood surface exhibited remarkable hydrophobic performance on different sections, with the water CA of cross section reaching 157.28°and tangential section reaching 141.53°. DI water droplet manifested an almost perfect spherical shape on the PDMS@wood surface, displaying hydrophobic properties on PDMS@wood. In addition, PDMS@wood retained excellent hydrophobic properties even after long-time storage and sandpaper abrasion test, demonstrating a great potential for large-scale production in industry.

## Supplementary Materials

The following are available online at https://www.mdpi.com/2073-4360/12/8/1757/s1. Video S1 (MOV): Dynamic Sliding Angle test process of PDMS@wood surface.
