# Supplementary material for: Novel Low-Temperature Chemical Vapor Deposition of Hydrothermal Delignified Wood for Hydrophobic Property
Source: Polymers (Basel). 2020 Aug 6;12(8):1757. doi: 10.3390/polym12081757 (PMC7463729; doi:10.3390/polym12081757)
Supplement: Supplementary file 1 [file polymers-12-01757-s001.zip › polymers-895516-supplementary.docx]

Supporting Information

Video S1 (MOV): Dynamic Sliding Angle test process of PDMS@wood surface
